# Bruton’s Tyrosine Kinase Inhibitors in Multiple Sclerosis: Mechanistic Considerations Across Relapsing and Progressive Disease

**DOI:** 10.3390/molecules31081272

**Published:** 2026-04-12

**Authors:** Qiying Ye, Siming Ma

**Affiliations:** 1 Department of Neurology, University of Minnesota, Minneapolis, MN 55455, USA; 2 Department of Medicine, MetroHealth Medical Center, Cleveland, OH 44109, USA; 3 Department of Medicine, Case Western Reserve University School of Medicine, Cleveland, OH 44106, USA

**Keywords:** BTK inhibitors, multiple sclerosis, relapsing multiple sclerosis, progressive multiple sclerosis, molecular design, pharmacokinetics, clinical trial interpretation

## Abstract

Multiple sclerosis (MS) reflects a dynamic interplay between peripheral immune activation and compartmentalized inflammation within the central nervous system (CNS). While current disease-modifying therapies effectively reduce relapse activity driven by transient peripheral immune infiltration, their impact on progressive disability remains limited, prompting interest in strategies targeting CNS-resident immune mechanisms. Bruton’s tyrosine kinase (BTK), expressed in B cells and myeloid-derived cells, including microglia, serves as a shared intracellular signaling node linking adaptive and innate immune pathways. Second-generation BTK inhibitors, including evobrutinib, tolebrutinib, fenebrutinib, remibrutinib, and orelabrutinib, have advanced through Phase II-III development in MS. These agents differ in binding mode, selectivity, pharmacokinetics, CNS penetration, and safety profiles, distinctions that may influence stage-specific therapeutic performance. Recent trials across relapsing and progressive phenotypes have yielded heterogeneous outcomes. Divergent signals in primary and secondary progressive MS reflect underlying biological heterogeneity and suggest that therapeutic responsiveness may depend on residual inflammatory activity, lesion biology, and pharmacologic characteristics. Emerging biomarker frameworks further emphasize the importance of stratifying inflammatory activity and degenerative progression when interpreting trial data. This review integrates molecular pharmacology and the most recent clinical evidence available through 2026 to examine how pharmacologic properties translate into stage-dependent therapeutic positioning. We also consider safety constraints within a disease-stage-specific benefit-risk framework, aiming to clarify the evolving role of BTK inhibition in MS.

## 1. Introduction

Multiple sclerosis (MS) is a chronic immune-mediated disorder of the central nervous system (CNS) characterized by inflammatory demyelination, progressive neuroaxonal injury, and neurodegeneration [1,2]. MS has traditionally been categorized into relapsing-remitting, secondary progressive, and primary progressive phenotypes for clinical and regulatory purposes [3,4]. However, increasing evidence supports viewing disease evolution along a biological continuum in which the relative contributions of acute peripheral immune infiltration and compartmentalized CNS inflammation shift over time [5,6,7]. As disease progresses along this continuum, pathological and imaging studies indicate that compartmentalized inflammation becomes increasingly prominent, manifesting as chronic active lesions and persistent microglial activation that may contribute to ongoing tissue injury [8,9]. These processes reflect a dynamic interplay between adaptive lymphocyte-driven responses and innate immune activation within the CNS [10,11]. Importantly, the relative contributions of CNS-resident inflammation and primary neurodegenerative mechanisms likely vary across disease stages and patient subgroups [5,12,13]. The stage-dependent and non-uniform nature of MS biology complicates therapeutic intervention. As dominant inflammatory mechanisms shift from peripheral adaptive immune activity toward compartmentalized CNS processes, therapies primarily targeting peripheral immunity may not uniformly address the drivers of progression [14,15,16,17]. This biological heterogeneity creates a therapeutic mismatch. While peripherally acting disease-modifying therapies successfully reduce relapse activity driven by adaptive immunity, they exert only modest effects on compartmentalized CNS inflammation that characterizes progressive disease and contributes to sustained disability accumulation [18,19,20,21,22].

Therapeutic strategies capable of modulating both peripheral B-cell activation and innate immune signaling within CNS myeloid populations may therefore offer a more integrated approach to stage-dependent disease biology [23]. Bruton’s tyrosine kinase (BTK) has emerged as a potential therapeutic target linking adaptive and innate immune pathways [24]. BTK is expressed in B cells as well as in myeloid-derived cells, including macrophages and CNS-resident microglia [25,26]. While B-cell receptor (BCR) signaling governs adaptive immune activation, Toll-like receptor (TLR) and Fcγ receptor pathways are prominently engaged within innate immune compartments; importantly, these distinct receptor systems converge on BTK as a shared intracellular signaling node [24]. Through this convergence, BTK integrates inflammatory cascades that regulate cytokine production, antigen presentation, and cellular activation across both peripheral and CNS-resident immune populations [27,28]. In this context, BTK inhibition represents a mechanistically grounded strategy to modulate convergent adaptive and innate immune signaling across peripheral and CNS compartments. This approach may be particularly relevant in disease stages characterized by persistent compartmentalized inflammation [29,30].

Recent phase II and III trials of BTK inhibitors in multiple sclerosis have yielded heterogeneous outcomes across relapsing and progressive phenotypes [31]. These divergent clinical signals raise important translational questions regarding whether BTK inhibition represents a uniform class effect or whether therapeutic performance may vary according to molecular design and compartmental target engagement. BTK inhibitors under development differ in binding mode, kinase selectivity, pharmacokinetic characteristics, and CNS penetration [32]. Although the extent to which these properties determine clinical efficacy remains uncertain, they provide a biologically plausible framework through which stage-specific differences in therapeutic response may be examined.

While recent reviews have summarized clinical trial outcomes of BTK inhibitors in multiple sclerosis, they have generally focused on efficacy and safety across compounds without explicitly integrating molecular pharmacology with stage-specific disease biology [1,22,27,29,32,33,34]. In this review, we integrate structural, signaling, and pharmacologic perspectives with the most recent Phase II and III clinical evidence to explore how distinct molecular features of BTK inhibitors might relate to divergent outcomes in relapsing and progressive MS.

## 2. BTK Structure, Signaling, and Mechanisms of Inhibition

BTK occupies a central position in both adaptive and innate immune signaling [35]. Understanding its structural domains, activation mechanisms, and downstream pathways is essential for interpreting how different inhibitors modulate immune responses across cell types. This section reviews BTK structure, cell-specific signaling, and preclinical evidence supporting its targeting in multiple sclerosis.

### 2.1. Structural Architecture of BTK

**Figure 1:**
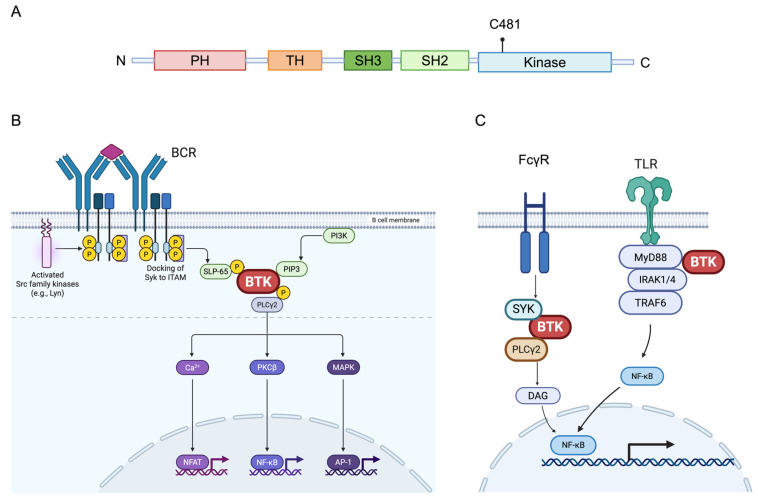
Domain architecture of Bruton’s tyrosine kinase and its signaling pathways in B cells and microglia. (**A**) Domain organization of BTK, comprising the pleckstrin homology (PH), Tec homology (TH), SH3, SH2, and kinase domains, with C481 located within the kinase domain. (**B**) In B cells, antigen-mediated B-cell receptor (BCR) engagement activates Src family kinases and SYK, leading to BTK recruitment, PLCγ2 activation, and downstream transcriptional responses. P indicates phosphorylation. (**C**) In myeloid cells and microglia, Fcγ receptor and Toll-like receptor signaling converge on BTK to modulate NF-κB–dependent inflammatory pathways. Created in Biorender. Ye, Q. (2026) https://BioRender.com/26016c8.

BTK is a member of the Tec family of non-receptor tyrosine kinases and plays a central role in B-cell and myeloid cell signaling [36,37]. Structurally, BTK comprises five conserved domains: an N-terminal pleckstrin homology (PH) domain, followed by a Tec homology (TH) region, SH3 and SH2 domains, and a C-terminal kinase domain [38] (Figure 1A). The PH domain mediates membrane recruitment through interaction with phosphatidylinositol (3,4,5)-trisphosphate (PIP3), enabling activation following receptor stimulation [39,40].

The catalytic activity of BTK resides in its C-terminal kinase domain, which contains the ATP-binding pocket responsible for substrate phosphorylation. A conserved cysteine residue at position 481 (Cys481) within the ATP-binding site permits covalent binding of irreversible BTK inhibitors [41]. This structural feature enables covalent BTK inhibitors to form an irreversible bond with Cys481, whereas reversible inhibitors bind non-covalently within the ATP-binding pocket without forming a covalent linkage. Subtle conformational differences within the ATP-binding site and surrounding residues influence inhibitor selectivity, off-target kinase interactions, and pharmacodynamic properties, making structural considerations central to therapeutic design [42,43]. These structural features not only determine inhibitor binding characteristics but also shape the extent and durability of downstream BTK signaling modulation within immune cells [44,45].

### 2.2. BTK Signaling in Peripheral Immune Cells

BTK functions as a critical signaling intermediary downstream of BCR engagement (Figure 1B). Upon antigen binding, phosphorylation of immunoreceptor tyrosine-based activation motifs (ITAMs) by Src-family kinases, most notably LYN, initiates recruitment of SYK and activation of phosphoinositide 3-kinase (PI3K), leading to generation of PIP3 at the plasma membrane [46,47]. Co-receptor CD19 amplifies PI3K signaling, further promoting membrane recruitment of BTK via its PH domain and enabling full kinase activation and subsequent phosphorylation of phospholipase Cγ2 (PLCγ2) [48,49].

Activated PLCγ2 generates inositol trisphosphate and diacylglycerol, triggering intracellular calcium mobilization and protein kinase C activation. These events converge on key transcriptional pathways, including NF-κB, MAPK, and NFAT signaling, which regulate B-cell survival, proliferation, cytokine production, and differentiation into memory or plasma cells [50,51]. Through its central position within the BCR cascade, BTK serves as a signaling amplifier of adaptive immune responses rather than a terminal effector, distinguishing BTK inhibition from strategies that directly deplete B cells [52]. In the context of multiple sclerosis, such modulation of peripheral B-cell activation is most directly associated with relapse biology, particularly in earlier disease stages characterized by active inflammatory infiltration [53].

### 2.3. BTK Signaling in Myeloid Cells and Microglia

Adaptive B-cell signaling and myeloid-driven innate activation are mechanistically intertwined in MS [13]. Examining these pathways in parallel, however, allows for clearer delineation of the distinct cellular environments in which BTK functions. In addition to its central role in B-cell receptor signaling, BTK is expressed in multiple myeloid-lineage cells, including macrophages, dendritic cells, and CNS-resident microglia [54]. In these cells, BTK participates in signaling cascades downstream of Fcγ receptors (FcγRs) and TLRs, where it contributes to innate immune activation [55] (Figure 1C).

Engagement of FcγRs by immune complexes induces phosphorylation of ITAMs, recruitment of SYK, and activation of BTK through PI3K-dependent generation of PIP3. Activated BTK promotes downstream phospholipase Cγ signaling, calcium mobilization, and activation of NF-κB and MAPK pathways, ultimately leading to transcription of pro-inflammatory mediators such as IL-1β, IL-6, and TNF [56,57]. Similarly, in TLR signaling, BTK interacts with adaptor proteins, including MyD88, to facilitate NF-κB activation and cytokine production [58,59]. BTK has also been implicated in the modulation of inflammasome activation, including NLRP3, in certain inflammatory contexts [60].

Microglia share many of these signaling mechanisms. In MS, chronic microglial activation is associated with smoldering lesions, cortical demyelination, and compartmentalized inflammation that persists behind a partially restored blood–brain barrier [61]. Unlike transient peripheral immune infiltration, microglial activation within chronic active lesions is spatially compartmentalized and temporally sustained, potentially maintaining inflammatory signaling even in the absence of overt clinical relapse [10,62]. The involvement of BTK in microglial signaling thus positions BTK inhibition at a convergence point between peripheral adaptive immunity and CNS-compartment innate activation- an axis increasingly implicated in progressive disease biology [55].

### 2.4. Mechanisms of BTK Inhibition

Pharmacologic inhibition of BTK primarily involves targeting the ATP-binding pocket within the kinase domain, thereby preventing phosphorylation of downstream substrates such as PLCγ2. Two principal classes of BTK inhibitors have been developed: covalent (irreversible) and reversible (non-covalent) inhibitors.

Covalent inhibitors form an irreversible bond with Cys481 within the ATP-binding site [42]. This covalent interaction results in sustained inhibition that persists until new BTK protein is synthesized, thereby extending pharmacodynamic effects beyond plasma half-life. Such prolonged target occupancy may permit lower systemic exposure while maintaining functional inhibition, but it also introduces considerations related to off-target kinase interactions and potential resistance mutations at Cys481 [41].

In contrast, reversible inhibitors bind reversibly within the ATP-binding cleft and stabilize inactive conformations of BTK through hydrogen bonding and hydrophobic interactions [63]. Their inhibitory effect depends on circulating drug concentration and equilibrium binding dynamics. Reversible inhibitors may retain activity in the presence of C481S mutations that disrupt covalent bonding and may offer advantages in terms of selectivity and safety [64]. Differences in binding mode influence kinase selectivity profiles, duration of target occupancy, dosing strategies, and potentially clinical tolerability.

Beyond simple kinase inhibition, pharmacologic properties such as blood–brain barrier penetration, half-life, and selectivity across the kinome may critically determine the therapeutic balance between peripheral immunomodulation and CNS-resident innate immune modulation [34,65,66]. As such, mechanistic differences among BTK inhibitors extend beyond shared target engagement and may shape stage-dependent therapeutic performance across relapsing and progressive MS phenotypes.

### 2.5. Preclinical Evidence Supporting BTK Inhibition in MS Models

Preclinical investigations have provided mechanistic support for BTK inhibition in experimental models of multiple sclerosis. In inflammatory demyelination paradigms, such as the experimental autoimmune encephalomyelitis (EAE), BTK inhibition was associated with attenuation of disease severity and modulation of adaptive immune responses. Rather than inducing broad B-cell depletion, BTK inhibitors have been shown to selectively limit pro-inflammatory B-cell differentiation while promoting regulatory B-cell properties, thereby reshaping antigen presentation and cytokine production without globally impairing B-cell function. In parallel, inhibition of BTK attenuated Fc receptor–dependent activation of myeloid cells and reduced pro-inflammatory cytokine release, supporting its role as a signaling intermediary linking adaptive and innate immune activation [55,67]

Beyond peripheral immune regulation, several studies have examined the impact of CNS-penetrant BTK inhibitors on innate immune signaling within the CNS. In chronic EAE paradigms and adoptive transfer models, BTK inhibition was associated with reduced microglial activation and diminished expression of pro-inflammatory mediators within the CNS, suggesting modulation of compartmentalized inflammatory processes. In toxin-induced demyelination models independent of peripheral adaptive immunity, BTK inhibition enhanced microglial clearance of myelin debris and accelerated remyelination, supporting a potential role in regulating CNS-resident repair dynamics [68]. In separate preclinical studies, remibrutinib exhibited efficacy in both B cell-dependent and myeloid-driven EAE models, with scRNA-seq analyses confirming reduced inflammatory activation in CNS-resident microglia [69].

Complementing these in vivo findings, transcriptomic analyses in murine and human microglial systems, including induced pluripotent stem cell-derived microglia and tri-culture models incorporating neurons and astrocytes, have identified BTK-dependent inflammatory gene signatures and modulation of Fcγ receptor-associated pathways. Collectively, these preclinical observations indicate that BTK inhibition can influence both adaptive and innate immune mechanisms relevant to MS pathobiology [55]

However, while animal and cellular models provide valuable mechanistic insights, they do not fully recapitulate the complexity and heterogeneity of progressive MS in humans. The extent to which modulation of microglial and compartmentalized inflammatory signaling observed in preclinical systems translates into sustained effects on disability progression remains to be determined.

## 3. Evolution of BTK Inhibitors: From Oncology to CNS-Targeted Agents

The development of BTK inhibitors has progressed from oncology-driven covalent kinase blockade toward agents specifically optimized for CNS inflammatory diseases [70]. This evolution reflects shifts in priorities: from maximizing durable target suppression in malignant B cells to achieving selective, sustained modulation of adaptive and innate immune signaling within the CNS [71].

### 3.1. First-Generation Covalent Inhibitors

Ibrutinib was the first-in-class covalent BTK inhibitor approved for the treatment of B-cell malignancies [72]. By irreversibly binding to Cys481 within the ATP-binding pocket, ibrutinib achieved prolonged BTK inhibition and demonstrated substantial clinical efficacy in chronic lymphocytic leukemia and related disorders [73,74,75]. This efficacy was established in landmark phase III randomized trials, including RESONATE, RESONATE-2, and ILLUMINATE, which demonstrated improved progression-free survival in patients with chronic lymphocytic leukemia compared with standard chemoimmunotherapy regimens [76,77,78].

However, first-generation inhibitors were characterized by limited kinase selectivity and off-target inhibition of kinases such as EGFR and ITK, contributing to adverse effects including bleeding and atrial fibrillation [79,80,81,82,83]. Beyond issues of selectivity, their development was primarily oriented toward systemic malignant B-cell suppression. Although ibrutinib demonstrates measurable CNS penetration and clinical activity in primary CNS lymphoma, it was not specifically optimized for sustained parenchymal exposure or modulation of compartmentalized neuroinflammation [84].

Thus, although ibrutinib established proof-of-concept for covalent BTK inhibition, its pharmacologic profile was not optimized for chronic inflammatory diseases of the CNS.

### 3.2. Second-Generation Covalent Inhibitors

Second-generation covalent BTK inhibitors, including acalabrutinib, zanubrutinib, and tirabrutinib, were developed to improve kinase selectivity while maintaining irreversible binding to Cys481 [85]. These agents demonstrated reduced off-target kinase inhibition and improved tolerability in hematologic indications [80,86,87]. Clinical efficacy of acalabrutinib was established in phase III trials, including ELEVATE-TN and ASCEND, which demonstrated improved progression-free survival compared with standard chemoimmunotherapy regimens in patients with chronic lymphocytic leukemia [87,88].

Despite these refinements, development efforts remained largely focused on hematologic malignancies, including systemic and CNS lymphomas, rather than on immune-mediated demyelinating disease [89,90]. Consequently, pharmacologic optimization for sustained CNS parenchymal exposure and modulation of resident innate immune signaling was not a primary design goal.

In addition to second-generation covalent inhibitors, a broader range of BTK inhibitors has been developed to address limitations of earlier compounds, including resistance mutations, selectivity profiles, and alternative immunologic indications [91,92]. These include non-covalent inhibitors and agents developed for autoimmune diseases, such as nemtabrutinib, rilzabrutinib, roczibrutinib, and docirbrutinib [93,94]. Notably, nemtabrutinib represents a next-generation non-covalent BTK inhibitor designed to overcome resistance mutations, such as Cys481 substitutions [95]. While these compounds represent important extensions of BTK inhibitor pharmacology, they do not fall within the conventional first- or second-generation covalent inhibitor framework and have not progressed into MS-specific clinical development.

### 3.3. CNS-Penetrant and MS-Focused BTK Inhibitors

The development of BTK inhibitors for multiple sclerosis marked a conceptual transition from oncology-driven kinase inhibition to CNS-directed immunomodulation. Although MS-directed BTK inhibitors share a common molecular target, they differ substantially in molecular binding characteristics, kinase selectivity profiles, and systemic pharmacokinetic and CNS distribution properties [29]. The following sections examine these distinctions sequentially: from molecular target engagement to kinase selectivity and finally to systemic pharmacokinetics and CNS exposure, to clarify how these properties may shape peripheral and central target engagement and ultimately influence clinical efficacy and safety across relapsing and progressive forms of MS.

#### 3.3.1. Binding Mode and Target Engagement

Among BTK inhibitors evaluated in MS, two principal functional classes can be distinguished based on binding mode: covalent, turnover-dependent inhibitors (e.g., evobrutinib, tolebrutinib, remibrutinib, orelabrutinib) and reversible, concentration-dependent inhibitors (e.g., fenebrutinib and BIIB091) [29]. Covalent inhibitors irreversibly bind to Cys481 within the ATP-binding site, resulting in sustained BTK inactivation that persists beyond the decline of plasma drug concentrations and depends on protein turnover for recovery [96,97]. In contrast, reversible inhibitors bind non-covalently within the kinase domain and exhibit equilibrium-dependent pharmacodynamics, such that their inhibitory effects depend on circulating drug concentrations and are lost upon drug removal [41,45]. Consistent with this distinction, comparative in vitro washout studies demonstrate rapid loss of cellular BTK inhibition following removal of non-covalent agents, whereas covalent inhibitors maintain suppression in a time-dependent manner [66].

These mechanistic differences may have implications beyond simple kinase inhibition. For example, CNS-penetrant covalent inhibitors such as tolebrutinib may be more likely to support sustained central target engagement, whereas reversible inhibitors such as fenebrutinib and BIIB091 depend on continuous systemic exposure to maintain BTK inhibition. These differences in binding mode may translate into distinct patterns of peripheral and CNS target engagement, although their clinical implications in relapsing and progressive MS remain to be fully established.

#### 3.3.2. Kinase Selectivity

Selectivity profiles vary substantially among BTK inhibitors and represent a major differentiating factor in their therapeutic development [66]. Structural similarities across kinase ATP-binding domains, particularly within the TEC family, create inherent challenges in achieving high specificity [98,99,100]. Consequently, the selectivity spectrum of each compound reflects both its chemical design and binding mode [101].

Beyond quantitative differences in kinase inhibition spectra, distinct BTK binding modes may also differentially influence downstream B-cell receptor signaling independent of catalytic suppression, suggesting that selectivity encompasses both kinome breadth and qualitative modulation of immune signaling pathways [102,103]

In the context of multiple sclerosis, long-term immune modulation in otherwise immunocompetent individuals necessitates a more refined selectivity profile. MS-focused BTK inhibitors were therefore engineered to improve kinase selectivity while maintaining adequate target occupancy. Nevertheless, meaningful differences in kinase inhibition spectra persist across compounds [104,105]. Variability in selectivity may influence safety signals, immunomodulatory breadth, and tolerability during chronic administration, particularly given the distinct balance required between suppressing pathogenic adaptive responses and preserving protective immune surveillance [106]. For example, earlier-generation inhibitors such as ibrutinib exhibit broader kinase inhibition profiles, whereas more recently developed agents demonstrate increased selectivity, potentially reducing off-target effects while preserving BTK-mediated immunomodulation. Similarly, among MS-directed BTK inhibitors, compounds such as tolebrutinib and remibrutinib exhibit more selective kinase profiles, whereas earlier agents may retain broader kinase inhibition spectra [29].

#### 3.3.3. Pharmacokinetics and CNS Penetration

Whereas the previous section addressed molecular binding characteristics, pharmacokinetic properties determine whether theoretical target engagement is achieved in vivo. Key parameters, including half-life, peak plasma concentration (Cmax), time to maximal concentration (Tmax), and overall systemic exposure (AUC), influence both the magnitude and duration of BTK inhibition at the organismal level, as detailed pharmacokinetic analyses have been reported elsewhere [32]. Although covalent inhibitors may exhibit pharmacodynamic persistence relative to plasma half-life, the extent of sustained in vivo inhibition varies across agents and depends on systemic exposure, protein binding, and tissue distribution [45,65].

In addition to systemic exposure, CNS penetration is a particularly important consideration in multiple sclerosis, especially in progressive disease characterized by compartmentalized inflammation. Emerging translational studies suggest that BTK inhibitors differ in their CNS pharmacokinetic profiles. Tolebrutinib was specifically engineered for enhanced CNS penetration, with molecular properties optimized for central exposure, and has demonstrated measurable cerebrospinal fluid concentrations that, in preclinical and early-phase studies, exceeded estimated pharmacodynamic thresholds [65,107]. In contrast, available data for evobrutinib indicate detectable but comparatively lower CSF exposure relative to in vitro inhibitory parameters, although the clinical significance of this observation remains uncertain [32]. Data for other agents remain limited or are primarily derived from conference presentations rather than peer-reviewed publications. Not all MS-directed BTK inhibitors were originally designed for CNS penetration, which may contribute to heterogeneity in clinical outcomes.

Differences in systemic pharmacokinetics, BTK occupancy kinetics, and CNS exposure may influence the relative balance between peripheral immune modulation and CNS-resident target engagement. However, cross-trial comparisons remain challenging due to heterogeneity in assay methodologies, timing of CSF sampling, and reporting standards.

#### 3.3.4. Individual Agents in Clinical Development

Evobrutinib was among the earliest BTK inhibitors evaluated in multiple sclerosis and represents a covalent, irreversible inhibitor targeting Cys481 within the ATP-binding pocket (Table 1). In first-in-human (phase I) studies, evobrutinib demonstrated rapid absorption (Tmax ~0.5 h) and a short plasma half-life (~2 h), yet achieved dose-dependent and durable BTK occupancy in peripheral blood mononuclear cells, consistent with irreversible target engagement. Sustained occupancy was observed beyond the plasma elimination phase, and no clinically relevant QT prolongation was detected [97]. In a randomized, placebo-controlled, double-blind phase II trial in patients with relapsing MS, with an open-label dimethyl fumarate (DMF) reference arm, evobrutinib was evaluated across multiple dose arms, with the primary endpoint of the cumulative number of gadolinium-enhancing lesions on MRI. Evobrutinib demonstrated reductions in gadolinium-enhancing lesions, supporting proof-of-concept for BTK modulation in inflammatory disease activity [108]. Post hoc analyses from the phase II program demonstrated dose-dependent reductions in slowly expanding lesion volume, an MRI marker associated with chronic active lesions [109]. In long-term extension analyses extending beyond 3.5 years of treatment, evobrutinib was detectable in cerebrospinal fluid at concentrations approximating free plasma levels, suggesting measurable CNS exposure [110]. However, in subsequent phase III trials comparing evobrutinib with teriflunomide, superiority in annualized relapse rate was not achieved [111] (Table 2). Collectively, these findings illustrate the evolving clinical and mechanistic profile of evobrutinib across development stages.

Tolebrutinib (PRN2246, SAR442168) is a covalent BTK inhibitor developed with enhanced CNS penetration to target compartmentalized inflammation. In first-in-human (phase I) studies, despite a short plasma half-life (~2 h), tolebrutinib achieved durable peripheral BTK occupancy consistent with irreversible binding, and cerebrospinal fluid exposure was confirmed [107]. In a randomized, double-blind phase IIb trial in patients with relapsing MS, tolebrutinib was evaluated across multiple dose arms, with the cumulative number of gadolinium-enhancing lesions on MRI as the primary endpoint. Tolebrutinib demonstrated dose-dependent reductions in new gadolinium-enhancing lesions, with the 60 mg dose showing the greatest effect [31]. A long-term extension suggested sustained MRI control and stable relapse rates over two years, although interpretation was limited by its open-label design [112]. Tolebrutinib subsequently advanced into Phase III programs across relapsing, secondary progressive, and primary progressive MS. In the HERCULES trial in nonrelapsing secondary progressive MS, tolebrutinib significantly reduced the risk of confirmed disability progression compared with placebo [113]. In contrast, in the PERSEUS study in primary progressive MS, tolebrutinib did not significantly reduce the risk of 6-month composite confirmed disability progression compared with placebo. However, treatment was associated with reduced brain volume loss over the study period, as reported in a late-breaking presentation at ACTRIMS 2026 [114].

Fenebrutinib is a highly selective, reversible, non-covalent BTK inhibitor designed to achieve potent target engagement while minimizing off-target kinase interactions. In early phase I studies, fenebrutinib was well tolerated, demonstrated dose-dependent BTK engagement, and exhibited a steady-state half-life of approximately 4–10 h, supporting once-daily dosing. Dedicated cardiac repolarization studies showed no clinically meaningful effects on the QT/QTc interval [115]. In the phase II FENopta trial, a randomized, double-blind study in patients with relapsing multiple sclerosis, with the cumulative number of gadolinium-enhancing lesions on MRI as the primary endpoint, fenebrutinib reduced new gadolinium-enhancing lesions, supporting progression into phase III development [116]. Fenebrutinib subsequently entered an extensive phase III program in both relapsing and progressive MS populations. In the Phase III FENtrepid trial in primary progressive MS, sponsor-reported topline results were presented at the ACTRIMS Forum 2026. These data indicated non-inferiority of fenebrutinib compared with ocrelizumab for 12-week composite confirmed disability progression [117]. In November 2025, sponsor-reported topline results indicated that one of two pivotal RMS trials (FENhance 2) met its primary endpoint of ARR reduction versus teriflunomide [118]. As full peer-reviewed datasets are not yet available, interpretation of these findings should be considered preliminary. Collectively, these findings position fenebrutinib as a reversible BTK inhibitor demonstrating clinical activity across relapsing and progressive disease spectra, including phase III assessment against an anti-CD20 comparator in progressive MS.

BIIB091 is a reversible, ATP-competitive BTK inhibitor that inhibits BTK signaling without covalent binding [119,120]. Preclinical studies demonstrate high kinase selectivity and potent target engagement. However, its physicochemical profile, including relatively high polarity, may be less favorable for CNS penetration compared with BTK inhibitors specifically optimized for CNS exposure [121]. This distinction may be relevant when considering potential differences in target engagement across peripheral and CNS compartments. The extent to which peripheral BTK inhibition translates into effective CNS target engagement remains uncertain. A Phase II randomized, active-controlled clinical study (FUSION; ClinicalTrials.gov identifier: NCT05798520) is currently ongoing to evaluate BIIB091 in relapsing multiple sclerosis, using a two-part design with diroximel fumarate (DRF) as an active comparator. The study primarily assesses safety, tolerability, and MRI measures of CNS inflammation, while also evaluating the effects of BIIB091 as monotherapy and in combination with DRF.

Remibrutinib and orelabrutinib represent additional covalent BTK inhibitors under investigation in multiple sclerosis. Remibrutinib has advanced directly into phase III evaluation in relapsing MS (REMODEL I/II), although detailed MS-specific phase II efficacy data are not available, likely reflecting cross-indication development and reliance on pharmacological data derived from other disease settings [122].

In contrast, orelabrutinib has reported Phase II MRI-based efficacy signals in relapsing MS at the ACTRIMS Forum 2025, demonstrating significant reductions in gadolinium-enhancing lesions relative to placebo, with further development ongoing [123]. Although both agents share irreversible binding to Cys481, differences in selectivity profiles, pharmacokinetic characteristics, and levels of clinical data maturity complicate direct comparison across compounds.

In addition, pirtobrutinib, a non-covalent BTK inhibitor originally developed for B-cell malignancies, had been evaluated in a planned Phase II randomized, placebo-controlled trial in relapsing multiple sclerosis (ClinicalTrials.gov identifier: NCT06104683) [124]. However, the study was subsequently withdrawn prior to initiation, and its role in MS remains uncertain.

Collectively, these pharmacologic distinctions suggest that BTK inhibitors should not be viewed as a uniform therapeutic class in MS. Instead, differences in binding mode, CNS exposure, and selectivity may contribute to the divergent clinical outcomes observed across compounds, particularly when evaluated in relapsing versus progressive disease.

## 4. Divergence of Clinical Outcomes Across Disease Stages

Despite shared mechanistic rationale, BTK inhibitors have demonstrated variable clinical performance across relapsing and progressive multiple sclerosis populations [33]. While early-phase studies suggested biological activity consistent with BTK-mediated modulation of B-cell and myeloid signaling, later-phase trials have revealed a more complex relationship between pharmacologic target engagement and clinical endpoints [125]. This divergence invites closer examination of disease stage-specific inflammatory biology, endpoint selection, and the therapeutic positioning of BTK inhibition within contemporary treatment paradigms.

### 4.1. Relapsing MS Trials

Relapsing multiple sclerosis has traditionally served as the primary setting for early-phase evaluation of novel disease-modifying therapies, owing to the availability of well-established inflammatory endpoints such as annualized relapse rate (ARR) and magnetic resonance imaging lesion activity [126,127,128]. These endpoints allow relatively rapid assessment of anti-inflammatory efficacy and have historically supported regulatory approval [129]. However, outcomes across Phase III programs have diverged, providing important insight into the translational limits of BTK pathway modulation in contemporary RMS populations [29,33].

Evobrutinib was the first BTK inhibitor to report Phase III RMS data. In the EVOLUTION RMS I and II trials, two parallel Phase III studies conducted in relapsing MS, evobrutinib was compared with the active comparator teriflunomide, an established disease-modifying therapy in RMS [130]. The primary endpoint was ARR. Evobrutinib did not demonstrate superiority over teriflunomide in ARR reduction [111]. Unlike teriflunomide, which limits proliferation of activated lymphocytes through DHODH inhibition, evobrutinib modulates B-cell receptor signaling without inducing broad lymphocyte depletion [131,132]. Importantly, these trials assessed relapse-driven endpoints in predominantly relapsing populations. Whether the broader adaptive-innate signaling modulation conferred by BTK inhibition translates into therapeutic differentiation in disease stages dominated by compartmentalized CNS inflammation remains to be determined.

Tolebrutinib likewise demonstrated reductions in inflammatory MRI activity in early-phase studies [31]. In the Phase III GEMINI 1 and 2 trials in relapsing MS, tolebrutinib was compared with the active comparator teriflunomide, with ARR as the primary endpoint. Tolebrutinib did not demonstrate superiority over teriflunomide in ARR reduction [133]. However, its development trajectory in RMS was complicated by temporary regulatory holds related to hepatic safety signals, which led to protocol adjustments. The extent to which these changes may have altered systemic exposure or target occupancy in relapse-focused trials remains to be clarified [134,135]. Importantly, relapse endpoints predominantly reflect peripheral inflammatory dynamics. Given the CNS-penetrant design of tolebrutinib and its capacity to modulate innate immune signaling within compartmentalized CNS environments, its therapeutic differentiation may not be fully captured by relapse-driven models alone.

Fenebrutinib, a reversible BTK inhibitor, has been evaluated in Phase III trials in relapsing MS. In the FENhance 1 and 2 trials, two randomized, double-blind, active-controlled studies, fenebrutinib was compared with the active comparator teriflunomide, with ARR as the primary endpoint. Sponsor-reported topline results from FENhance 2 indicated a reduction in ARR compared with teriflunomide, although full peer-reviewed data are not yet available [118].

Taken together, RMS trials of BTK inhibitors reveal a consistent ability to modulate inflammatory MRI activity, but variable success in outperforming established therapies on relapse endpoints. In contemporary RMS cohorts characterized by declining baseline relapse rates and active comparator designs, the therapeutic window for incremental relapse suppression may be limited [136,137]. In addition, relapse endpoints primarily capture peripheral adaptive immune activity and may not fully reflect the CNS-directed and innate immune effects of BTK inhibition. Divergent signals across RMS and progressive MS populations may ultimately provide insight into whether pharmacologic modulation of BTK differentially impacts stage-specific pathogenic mechanisms, rather than representing a uniform class effect. These observations have prompted growing interest in evaluating BTK inhibitors in progressive MS, where CNS-compartmentalized inflammation may represent a more relevant therapeutic target.

### 4.2. Progressive MS Trials: Testing the CNS-Compartment Hypothesis

Relapse-focused trials primarily interrogate mechanisms driving acute peripheral inflammatory activity [138]. However, the CNS-compartment hypothesis posits that intrathecal immune signaling, including chronic microglial activation and smoldering lesion biology, may contribute more substantially to disability progression in progressive MS [10,139]. If BTK inhibition exerts clinically meaningful effects through modulation of convergent adaptive-innate signaling within the CNS, such effects may be more discernible in progressive populations than in relapse-dominated cohorts. Importantly, progressive MS does not represent a uniform biological state [140,141,142]. The relative contributions of residual inflammatory activity and established neurodegenerative processes vary across phenotypes and disease stages [143]. Progressive trials therefore function not as a single test of the CNS-compartment hypothesis, but as a series of biological experiments conducted across heterogeneous disease contexts.

#### 4.2.1. Clinical Signals from Progressive Trials

Within this framework, divergent outcomes across progressive trials acquire mechanistic relevance. Among BTK inhibitors, tolebrutinib has provided the most informative data in progressive populations. In the Phase III HERCULES trial, a randomized, placebo-controlled study in non-relapsing SPMS, treatment was associated with a reduction in 6-month confirmed disability progression (CDP), a composite endpoint reflecting sustained disability worsening, compared with placebo [113]. Because this population minimizes relapse-driven inflammatory activity, the observation of a disability signal is conceptually notable and has been interpreted as supportive of activity beyond suppression of acute peripheral immune infiltration.

In contrast, sponsor-reported preliminary data from the Phase III PERSEUS trial, a randomized, double-blind, placebo-controlled study in patients with PPMS, with 6-month confirmed disability progression as the primary endpoint, indicated no statistically significant difference between tolebrutinib and placebo, although reductions in brain volume loss were observed [114]. These divergent findings across progressive phenotypes suggest that disability biology in SPMS and PPMS may not be uniform and that the CNS-compartment hypothesis may not operate equivalently across all progressive subgroups, and therapeutic responsiveness may depend on underlying lesion biology, inflammatory burden, and disease stage.

Although tolebrutinib currently provides the most mature progressive data within the BTK inhibitor class, other agents are undergoing evaluation in progressive populations. Fenebrutinib has been studied in the Phase III FENtrepid trial, a randomized, double-blind, active-controlled study in patients with PPMS comparing fenebrutinib with ocrelizumab, with CDP as the primary endpoint. Sponsor-reported preliminary results presented at the ACTRIMS Forum 2026 indicated non-inferiority to ocrelizumab on CDP at 12 weeks [117]. While durability at 24-week confirmation and full peer-reviewed datasets remain awaited, these findings introduce a potentially important inflection point in assessing the role of reversible BTK inhibition in progressive disease. Whether comparable performance relative to an established anti-CD20 therapy reflects effective modulation of CNS-resident immune mechanisms, peripheral immune control, or a combination of both remains to be clarified.

Taken together, progressive trial outcomes suggest that BTK inhibition may exert measurable effects in selected progressive subpopulations, but efficacy has not been uniform across phenotypes, likely reflecting underlying biological heterogeneity and the need for more precise patient stratification.

#### 4.2.2. Biological Heterogeneity in Progressive MS

The divergent signals observed across progressive phenotypes suggest the biological heterogeneity of progressive MS itself. Disability progression in progressive MS reflects a dynamic interplay among persistent compartmentalized inflammation, axonal loss, mitochondrial dysfunction, and age-related neurobiological vulnerability [10]. In some patients, residual inflammatory signaling remains biologically active and potentially modifiable. In others, neurodegenerative cascades may predominate, limiting the impact of immune-directed therapies [62].

Although SPMS and PPMS share a largely overlapping pathological framework, several distinctions have been described that may contribute to differential therapeutic responsiveness [144]. Pathological studies, including post-mortem autopsy analyses, demonstrate that SPMS is associated with a higher degree of inflammatory activity, including persistent meningeal inflammation and ectopic lymphoid follicle-like structures, which have been reported in approximately 40–70% of cases [145,146]. In contrast, PPMS exhibits fewer inflammatory cells within lesions and perivascular regions on histopathological examination, indicating a relatively lower level of overt inflammatory activity [147,148]. Imaging studies provide complementary evidence at the structural level. SPMS is more often associated with larger and more confluent lesions, whereas PPMS typically shows fewer, smaller, and more diffusely distributed lesions, consistent with a more diffuse pattern of tissue injury [149,150].

Despite these differences, both phenotypes converge on shared mechanisms of compartmentalized CNS inflammation, mitochondrial dysfunction, and neurodegeneration, suggesting that distinctions are primarily quantitative rather than qualitative [151]. Such heterogeneity implies that therapeutic efficacy cannot be interpreted solely through aggregate trial outcomes. Instead, clinical benefit may emerge preferentially in subgroups enriched for modifiable inflammatory substrates. The apparent divergence between SPMS and PPMS outcomes may therefore reflect differences in lesion activity, inflammatory compartmentalization, and stage-specific biology rather than intrinsic inconsistency in BTK inhibition as a mechanistic strategy. This framework may partially explain why BTK inhibition, which targets B-cell-mediated and innate immune signaling, could demonstrate greater therapeutic impact in progressive subpopulations with residual inflammatory activity, such as SPMS, while showing more limited efficacy in inflammation-poor phenotypes such as PPMS.

#### 4.2.3. The Role of Biomarker-Guided Interpretation

Beyond disease biology, interpretation of progressive trials is further shaped by the current limitations and evolving roles of fluid biomarkers in stratifying disease activity [152,153]. Among available fluid biomarkers, serum neurofilament light chain (sNfL) has emerged as a robust marker of neuroaxonal injury and is strongly associated with acute inflammatory activity [154]. It performs well as a biomarker of relapse activity and treatment response in relapsing MS [155,156]. In contrast, its ability to capture disability progression independent of relapse activity appears more limited in progressive disease. The differential behavior of sNfL across relapsing and progressive phenotypes itself underscores the stage-dependent biology of MS [157].

By contrast, serum glial fibrillary acidic protein (sGFAP), reflecting astroglial activation and chronic glial pathology, has been more consistently linked to progressive phenotypes and subsequent disability worsening [158,159,160]. Elevated sGFAP levels have been linked to gray matter atrophy and future confirmed disability worsening, even in the absence of overt inflammatory relapse [161]. In progressive-enriched cohorts, higher sGFAP, but not sNfL, predicted future confirmed disability progression, and the prognostic effect of sGFAP appeared strongest in patients with low sNfL levels [162]. These findings suggest that sGFAP and sNfL capture partially complementary biological dimensions, corresponding broadly to degenerative and inflammatory axes of disease, respectively [163].

Importantly, combined assessment of sNfL and sGFAP may provide a more refined framework for biological stratification. Patients with elevated sNfL but low sGFAP may reflect predominantly inflammatory disease activity, whereas those with elevated sGFAP and low sNfL may represent a more degeneration-driven phenotype [163]. Such distinctions may be particularly relevant for interpreting heterogeneous treatment responses in progressive MS trials, especially for therapies targeting CNS-compartmentalized inflammation.

These observations reinforce the notion that progressive MS encompasses overlapping but distinct pathological processes, including residual inflammatory activity, chronic glial activation, and neurodegeneration. Current biomarker strategies may therefore incompletely capture the compartmentalized inflammatory dynamics that BTK inhibitors are hypothesized to target. In this context, progressive MS trials may represent not a single test of the CNS-compartment hypothesis, but multiple biological experiments conducted across heterogeneous disease states defined by differing contributions of inflammation and degeneration. Recent multimodal approaches integrating metabolomic and protein biomarkers further improve long-term prediction of progression, reinforcing the concept that progression reflects intersecting inflammatory, glial, and metabolic pathways [164].

#### 4.2.4. Conceptual Implications

Taken together, progressive BTK inhibitor trials neither conclusively confirm nor refute the CNS-compartment hypothesis; rather, they redefine its therapeutic boundaries. Signals observed in non-relapsing SPMS suggest that intrathecal immune modulation may influence disability progression under specific biological conditions. However, variability across progressive phenotypes indicates that therapeutic responsiveness likely depends on the presence of modifiable inflammatory substrates rather than representing an inherent class-wide effect.

Importantly, clinical endpoints in MS integrate multiple biological dimensions. Annualized relapse rate primarily reflects overt inflammatory events, whereas confirmed disability progression captures a composite of inflammatory burden, axonal reserve, compensatory capacity, and age-related vulnerability [127]. These measures are related but not interchangeable. Consequently, absence of dramatic relapse superiority does not necessarily imply lack of biologic activity, just as modest disability signals in progressive disease do not establish universal mechanistic validation.

The reported inclusion of fenebrutinib in a Phase III PPMS program, with sponsor-described non-inferiority to ocrelizumab, further challenges simplistic mechanistic interpretations [117]. If confirmed, such findings would suggest that both peripheral B-cell suppression and intrathecal immune modulation may contribute to clinical benefit, and that reversible and covalent BTK inhibition cannot be interpreted solely through a single mechanistic lens.

Collectively, divergent outcomes across BTK inhibitor programs reflect the intersection of molecular pharmacology, disease-stage biology, and trial design. Rather than framing these differences as binary indicators of class success or failure, a more nuanced interpretation recognizes that therapeutic positioning increasingly depends on aligning pharmacologic properties with the dominant biological drivers present in a given disease context. Ongoing trials across well-characterized subgroups will be critical in defining the precise role of BTK inhibition within the evolving treatment paradigm.

## 5. Safety and Pharmacologic Considerations

### 5.1. Hepatic Safety and Regulatory Considerations

Hepatic safety has emerged as an important consideration in the development of BTK inhibitors for multiple sclerosis. Across several agents, asymptomatic elevations in alanine aminotransferase (ALT) and aspartate aminotransferase (AST) have been reported, consistent with small-molecule hepatic metabolism and potential off-target kinase interactions [106]. In most programs, these laboratory abnormalities were reversible and manageable with structured monitoring protocols [111,116].

Tolebrutinib, however, has demonstrated a more substantial hepatotoxic signal. In 2022, clinical studies were placed under partial regulatory hold following cases of drug-induced liver injury (DILI), including events meeting Hy’s Law criteria [135]. Although trials resumed with intensified monitoring strategies, subsequent regulatory review identified multiple Hy’s Law cases, including one fatal outcome following liver transplantation. In late 2025/early 2026, regulatory feedback indicated that a favorable benefit–risk profile had not been established, driven by concerns related to severe idiosyncratic hepatotoxicity and uncertainty regarding efficacy. No clinically identifiable subgroup was identified in which the potential benefits were considered to outweigh these risks [165,166].

These developments indicate heterogeneity in hepatic risk across the BTK inhibitor class [167]. Mild transaminase elevations have been reported in several programs; however, clinically significant hepatotoxicity and regulatory consequences have varied across agents. Whether hepatotoxicity reflects covalent binding properties, metabolite formation, dose-dependent exposure, or patient-specific susceptibility remains incompletely defined.

Nevertheless, benefit–risk interpretation in multiple sclerosis is inherently stage-dependent [168]. Progressive phenotypes differ in projected disability trajectory, therapeutic alternatives, and clinical urgency. In primary progressive MS, where treatment options remain limited and disability accrues steadily, the threshold for acceptable risk may be interpreted differently than in secondary progressive or relapse-dominant contexts with available alternatives. Accordingly, identical safety signals may carry distinct therapeutic implications depending on disease stage and available comparators.

These safety considerations are particularly relevant in the context of long-term administration, where chronic exposure may increase cumulative toxicity risk and impose constraints on dose optimization and treatment continuity. Consistent with experience from other BTK inhibitor indications, treatment-emergent adverse events may affect long-term tolerability and, in some cases, lead to treatment discontinuation [73,106]. In addition, sustained monitoring strategies may be required to detect delayed or subclinical hepatic injury during prolonged treatment. Ongoing pharmacovigilance and long-term safety characterization will be essential in determining whether hepatic risk represents an agent-specific liability or a broader constraint of sustained BTK inhibition.

### 5.2. Reproductive and Postpartum Considerations in Women of Childbearing Age

Multiple sclerosis predominantly affects women during their reproductive years, making pregnancy planning central to long-term therapeutic decision-making [169,170]. Pregnancy is associated with reduced relapse activity, whereas the postpartum period carries increased inflammatory risk, necessitating careful coordination of treatment interruption and re-initiation [171,172,173,174].

Established high-efficacy therapies present distinct reproductive considerations that reflect differences in pharmacokinetics, mechanism of action, and fetal exposure risk [175,176,177,178]. Injectable platform therapies such as interferon beta and glatiramer acetate have relatively favorable safety profiles and may be continued in selected cases [179,180]. In contrast, oral small-molecule agents, including sphingosine-1-phosphate receptor modulators and teriflunomide, are contraindicated due to teratogenic risk and require defined washout or elimination procedures prior to conception [181]. Monoclonal antibodies, including anti-CD20 therapies, introduce additional complexity, as prolonged pharmacodynamic effects and transplacental transfer in later trimesters may result in fetal immune alterations despite limited early exposure [175,176,182]. More broadly, most therapies require individualized planning of treatment interruption and re-initiation, balancing maternal disease control against fetal safety [183,184].

BTK inhibitors represent a distinct pharmacologic category. As orally administered small molecules with relatively short systemic half-lives and without sustained lymphocyte depletion, they may theoretically allow more flexible treatment discontinuation strategies prior to conception and earlier postpartum re-initiation. However, no peer-reviewed human pregnancy outcome data are currently available for BTK inhibitors in MS. Information regarding placental transfer, teratogenicity, and neonatal immune effects remains limited, and pregnant or breastfeeding individuals have been excluded from clinical trials [33,185]. Accordingly, BTK inhibitors cannot presently be considered pregnancy-compatible therapies.

Breastfeeding introduces additional uncertainty. Data regarding excretion of BTK inhibitors into human milk are lacking, and the potential impact of low-level infant exposure on immune development is unknown [186]. In contrast, limited data from other therapies such as interferon beta and glatiramer acetate suggest minimal transfer into breast milk and no adverse infant outcomes [187,188]. Until prospective registries and post-marketing data mature, reproductive use of BTK inhibitors should be approached cautiously with individualized risk–benefit assessment and multidisciplinary counseling.

## 6. Future Perspectives in BTK-Targeted CNS Therapy

### 6.1. Optimization of Molecular Design

BTK inhibitors were initially developed in oncology and later adapted for autoimmune and CNS-directed indications, with each stage of development involving progressive molecular refinement. Early covalent inhibitors established proof-of-concept for pathway modulation, whereas subsequent development has emphasized balancing potency, kinase selectivity, pharmacokinetic stability, tissue penetration, and long-term tolerability [34,70].

Reversible BTK inhibitors that do not rely on covalent interaction with the C481 residue have been explored to address resistance mechanisms identified in oncology [189,190]. In autoimmune disease, reversibility may theoretically allow more tunable pathway modulation and reduced sustained off-target engagement. However, any potential differentiation must ultimately be evaluated within the therapeutic window required for chronic treatment in relatively young populations.

Optimization of CNS penetration remains a central design objective, particularly under the CNS-compartment hypothesis. Yet achieving sufficient brain exposure while preserving systemic safety presents a complex medicinal chemistry challenge. Enhanced CNS availability does not inherently translate into superior clinical outcomes, and may narrow safety margins if systemic exposure thresholds are exceeded [65].

Emerging structural studies suggest that BTK may exert kinase activity-independent scaffolding functions, raising the possibility that catalytic inhibition alone may not fully capture the biological consequences of BTK modulation [191]. Whether such non-catalytic roles meaningfully contribute to MS pathobiology remains uncertain, but these observations highlight that molecular refinement must align mechanistic targeting with tolerability constraints.

Future optimization will likely focus not on maximal pathway suppression, but on refining structure–function–exposure relationships to achieve stage-appropriate immunomodulation within an acceptable long-term safety profile.

### 6.2. Broader Implications in Neuroimmune and Neurodegeneration Disorders

Beyond multiple sclerosis, BTK signaling has attracted interest in CNS disorders characterized by chronic innate immune activation [192,193]. In Alzheimer’s disease (AD), microglial activation is increasingly recognized as a driver of amyloid- and tau-associated neurodegeneration [194,195,196,197]. BTK has been shown to be expressed in microglia and upregulated in both transgenic AD mouse models and postmortem human AD brain tissue, supporting its potential involvement in disease-associated neuroinflammation [198]. Experimental inhibition of BTK attenuates PLCγ2 signaling, reduces microglia-mediated synaptic phagocytosis, and mitigates downstream neuroinflammatory cascades, resulting in preservation of synaptic structure and improved behavioral performance in rodent models [193]. These findings suggest that BTK may participate in innate immune pathways contributing to neurodegenerative progression.

However, the majority of available data in AD and other neurological disorders derive from preclinical studies, frequently involving first-generation BTK inhibitors such as ibrutinib, which differ in kinase selectivity and CNS pharmacokinetic properties from agents currently under development for MS [192]. Direct extrapolation across compounds or disease contexts should therefore be approached cautiously.

Beyond AD, mechanistic hypotheses implicating BTK signaling have been proposed in other neuroimmune-associated disorders, including Parkinson’s disease and ischemic brain injury. Nevertheless, pharmacologic validation and clinical translation in these settings remain limited. At present, extension of BTK-targeted strategies beyond MS should be regarded as hypothesis-generating rather than clinically established.

## 7. Conclusions

Bruton’s tyrosine kinase inhibition represents a mechanistically grounded strategy aimed at bridging adaptive B-cell signaling and innate myeloid-driven inflammation in multiple sclerosis. By targeting pathways relevant to both peripheral immune activation and CNS-resident microglial signaling, BTK inhibitors have been positioned as potential modulators of relapse biology as well as compartmentalized inflammatory processes implicated in progressive disease.

Clinical development has underscored the complexity of translating this dual-mechanism rationale into consistent therapeutic benefit. Heterogeneous outcomes across phase III programs highlight that BTK inhibitors cannot be regarded as a uniform class; rather, structural properties, kinase selectivity, and CNS penetration profiles likely determine the extent of intrathecal target engagement achieved.

Future progress will depend on integrating pharmacologic precision with mechanistic biomarkers capable of capturing CNS-compartmentalized inflammation and disability progression. Whether BTK inhibition ultimately reshapes the therapeutic landscape of progressive MS will hinge on demonstrating sustained effects beyond relapse suppression and into the domain of neurodegenerative trajectory modification.

## Figures and Tables

**Table 1:** Binding mode, selectivity, CNS penetration, PK/PD characteristics, and clinical implications of representative BTK inhibitors.

Compound	Binding Mode	Selectivity Profile	CNS Penetration	PK/PD Characteristics	Clinical Implication
Ibrutinib (PCI-32765)	Covalent (irreversible)	Broad (multiple off-target kinases)	Moderate (not CNS-optimized)	Sustained, turnover-dependent	Primarily systemic; limited CNS targeting
Acalabrutinib (ACP-196)	Covalent (irreversible)	Moderate (reduced off-target activity)	Low-moderate (limited data)	Sustained, improved selectivity	Peripheral modulation; reduced off-target effects
Zanubrutinib (BGB-3111)	Covalent (irreversible)	Moderate-high (improved selectivity)	Unclear/limited data	Sustained, high occupancy	Hematologic focus; CNS relevance unclear
Tirabrutinib (ONO-4059)	Covalent (irreversible)	High (limited off-target activity)	Moderate (CNS lymphoma evidence)	Sustained, selective	CNS activity in oncology; MS relevance uncertain
Nemtabrutinib	Non-covalent (reversible)	Moderate-high (broad kinase profile)	Unclear/limited data	Reversible, concentration-dependent; active against Cys481 mutations	Designed to overcome resistance; primarily hematologic, CNS relevance unclear
Evobrutinib	Covalent (irreversible)	High (improved specificity)	Low-moderate	Sustained, partial exposure dependence	Likely peripheral-dominant effects
Fenebrutinib (GDC-0853)	Non-covalent (reversible)	High (highly selective)	Moderate (exposure-dependent)	Reversible, concentration-dependent	Requires continuous exposure; CNS effects limited
Tolebrutinib	Covalent (irreversible)	High	High (CNS-optimized)	Sustained, CNS-penetrant	Potential CNS-compartment targeting (progressive MS)
Orelabrutinib	Covalent (irreversible)	High (minimal off-target activity)	Unclear/limited data	Sustained, selective	Favorable safety; CNS role unclear
Remibrutinib	Covalent (irreversible)	High (highly selective)	Limited (not CNS-optimized)	Potent, sustained	High selectivity; CNS effects under investigation
BIIB091	Non-covalent (reversible)	High	Limited (predicted)	Reversible, concentration-dependent	Combination strategy (±DRF); clinical role under evaluation

**Table 2:** Key Phase II and III clinical trials of BTK inhibitors in multiple sclerosis and their primary outcomes.

Drug	Trial Name	Phase	n	Population	Comparator	Duration	Primary Endpoint	Key Outcome
Evobrutinib	Phase II study	II	~260	RMS	Placebo (with DMF reference)	24–48 w	Gd lesions	Reduced lesions
	EVOLUTION I/II	III	~2290	RMS	Teriflunomide	~156 w	ARR	Not superior
Tolebrutinib	Phase IIb	II	130	RMS	Placebo	16 w	Gd lesions	Reduced lesions
	GEMINI 1/2	III	~1870	RMS	Teriflunomide	~139 w	ARR	Not superior
	HERCULES	III	~1130	non-relapsing SPMS	Placebo	~133 w	CDP	Reduced progression
	PERSEUS	III	—	PPMS	Placebo	Not reported	CDP	Not met (sponsor-reported)
Fenebrutinib	FENopta	II	~110	RMS	Placebo	12 w	Gd lesions	Reduced lesions
	FENhance 2	III	~1500	RMS	Teriflunomide	≥96 w	ARR	Reduced ARR (sponsor-reported, topline)
	FENtrepid	III	~985	PPMS	Ocrelizumab	≥120 w	CDP	Non-inferior (sponsor-reported, preliminary)
Orelabrutinib	Phase II study	II	~158	RMS	Placebo	24 w	Gd lesions	Reduced lesions (conference report)
Remibrutinib	REMODEL 1/2	III	—	RMS	Teriflunomide	Not reported	ARR	Ongoing
BIIB091	FUSION	II	—	RMS	DRF	~48 w	Safety and MRI measures	Not yet reported

## Data Availability

No new data were created or analyzed in this study.

## Abbreviations

The following abbreviations are used in this manuscript:

MS	Multiple sclerosis
CNS	Central nervous system
BTK	Bruton’s tyrosine kinase
BTKi	Bruton’s tyrosine kinase inhibitor
TLR	Toll-like receptor
BCR	B-cell receptor
PH	Pleckstrin homology
TH	Tec homology
PIP3	Phosphatidylinositol (3,4,5)-trisphosphate
ITAM	Immunoreceptor tyrosine-based activation motifs
PI3K	Phosphoinositide 3-kinase
PLCγ2	Phospholipase Cγ2
FCγR	Fcγ receptors
sNFL	Serum neurofilament light chain
sGFAP	Serum glial fibrillary acidic protein
ALT	Alanine aminotransferase
AST	Aspartate aminotransferase
DILI	Drug-induced liver injury
ARR	Annualized relapse rate
